# Analytic Approximate Solution for Falkner-Skan Equation

**DOI:** 10.1155/2014/617453

**Published:** 2014-04-30

**Authors:** Vasile Marinca, Remus-Daniel Ene, Bogdan Marinca

**Affiliations:** ^1^Department of Mechanics and Vibration, Politehnica University of Timi*ș*oara, 300222 Timi*ș*oara, Romania; ^2^Department of Electromechanics and Vibration, Center for Advanced and Fundamental Technical Research, Romania Academy, 300223 Timi*ș*oara, Romania; ^3^Department of Mathematics, Politehnica University of Timi*ș*oara, 300006 Timi*ș*oara, Romania; ^4^Department of Applied Electronics, Politehnica University of Timi*ș*oara, 300223 Timi*ș*oara, Romania

## Abstract

This paper deals with the Falkner-Skan nonlinear differential equation. An analytic approximate technique, namely, optimal homotopy asymptotic method (OHAM), is employed to propose a procedure to solve a boundary-layer problem. Our method does not depend upon small parameters and provides us with a convenient way to optimally control the convergence of the approximate solutions. The obtained results reveal that this procedure is very effective, simple, and accurate. A very good agreement was found between our approximate results and numerical solutions, which prove that OHAM is very efficient in practice, ensuring a very rapid convergence after only one iteration.

## 1. Introduction

It is known that the word “viscoelastic” means the simultaneous existence of viscous and simultaneous elastic responses of a material. Some materials having a viscoelastic behavior are relevant in many fields of study for industrial and technological applications such as polymers, plastic processing, cosmetics, geology composites, paint flow, adhesives, towers generators, accelerators, electrostatic filters, droplet filters, and the design of heat exchanges [1].

Motivated by significant applications of viscoelastic materials, a substantial amount of research works has been invested in the study of nonlinear systems. In 1931, Falkner and Skan [2] have used some approximate procedures to solve boundary-layer equations. Hartree [3] found the numerical solution using a shooting method with *F*′′(0) (see (7)) as free parameter. The boundary conditions (8) arise in the study of viscous flow past a wedge of angle *βπ*; *β* > 0 corresponds to flow toward the wedge and *β* < 0 corresponds to flow away from the wedge. The special case *β* = 0  is called the Blasius equation where the wedge reduced to a flat plate. In [4, 5], it is proved that if 0 ≤ *β* ≤ 1 then the Falkner-Skan equation (7) with initial conditions (8) admits a unique smooth solution. For −0.1988 < *β* < 0 there exist two solutions, that is, one with  *F*′′(0) > 0  and the other one with *F*′′(0) < 0. Botta et al. [6] showed that the solution of Falkner-Skan equation is unique for *β* > 1 under the restriction 0 < *F*′(0) < 1. Forced convection boundary-layer flow over a wedge with uniform suction or injection is analyzed by Yih [7]. Asaithambi [8] studied the Falkner-Skan equation using finite difference scheme. In [9], Zaturska and Banks presented a new solution branch in function of parameter *β*. This solution branch is found to end singularity at *β* = 1; its structure is analytically investigated and the principal characteristics are described. Also the spatial stability of such solutions is commented on. The differential transformation is adopted to investigate the velocity and shear-stress fields associated with Falkner-Skan boundary-layer problem in [10]. A group of transformations is used to reduce the boundary value problem into a pair of initial value problems, which are then solved by means of the differential transformation method. The nonlinear ordinary differential equation is solved using Adomian decomposition method (ADM) by Elgazery [11] such that the condition at infinity was applied to a related Padé approximation and Laplace transformation to the obtained solution. Also ADM is used in [12] by Alizadeh et al. to find an analytical solution in the form of infinite power series. Magnetohydrodynamic effects on the Falkner-Skan wedge flow are studied by Abbasbandy and Hayat in [13]. The same authors used Hankel-Padé and homotopy analysis method for the derivation of the solutions [14]. From a fluid mechanical point of view, the pathophysiological situation in myocardical bridges involves fluid flow in a time dependent flow geometry caused by contracting cardiac muscles overlying an intramural segment of the coronary artery. A boundary-layer model for the calculation of the pressure drop and flow separation is presented in [15] under the assumption that the idealized flow through a constriction is given by near equilibrium velocity profiles of the Falkner-Skan-Cooke family, the evolution of the boundary-layer is obtained by the simultaneous solution of the Falkner-Skan equation and the transient non-Kármán integral momentum equation.

Pirkhedri et al. [16] developed a numerical technique transforming the governing partial differential equation into a nonlinear third-order boundary value problem by similarity variables and then solved it by the rational Legendre collocation method. It used transformed Hermite-Gauss nodes as interpolation points. The steady Falkner-Skan solution for gravity-driven film flow of micropolar fluid is investigated in [17]. The ordinary differential equations are solved numerically using an implicit finite difference scheme known as the Keller-box method. In [18], Lakestani truncated the semi-infinite physical domain of the problem to a finite domain expanding the required approximate solution as the elements of Chebyshev cardinal functions. Yun proposed in [19] an iterative method for solving the Falkner-Skan equation in the form of polynomial series without requiring any differentiations or integrations of the previous iterate solutions. The author suggests a correction method which is compared with the successive differences of the iterations. In [20], Hendi and Hussain considered Falkner-Skan flow over a porous surface taking into account the case of uniform suction/blowing. Stream function formulation and suitable transformations reduce the arising problem to ordinary differential equation which has been solved by homotopy analysis method.

In science and engineering there exist a lot of nonlinear differential equations and even strongly nonlinear problems which are still very difficult to solve analytically by using traditional methods. Many methods exist for approximating the solutions of nonlinear problems, for example, the Adomian decomposition method [21], the modified Lindstedt-Poincare method [22], the parameter-expansion method [23], optimal variational method [24], optimal homotopy perturbation method [25], and so on [26].

The aim of the present paper is to propose an accurate approach to Falkner-Skan equation using an analytical technique, namely, optimal homotopy asymptotic method [26–28].

The validity of our procedure, which does not imply the presence of a small parameter in the equation, is based on the construction and determination of the auxiliary functions combined with a convenient way to optimally control the convergence of the solution. The efficiency of the proposed procedure is proved while an accurate solution is explicitly analytically obtained in an iterative way after only one iteration.

## 2. The Governing Equation

The two-dimensional laminar boundary-layer equations of an incompressible fluid subject to a pressure gradient are [2, 3, 9, 12]

(1) $$\begin{array}{c} u\frac{\partial u}{\partial x}+v\frac{\partial u}{\partial y}=-\frac{1}{\rho }p^{\prime }+\nu \frac{\partial^{2}u}{\partial y^{2}}, \end{array}$$

(2) $$\begin{array}{c} \frac{\partial u}{\partial x}+\frac{\partial v}{\partial y}=0, \end{array}$$

where *p*′ is the pressure gradient, *p*′ = −*ρU*(∂*U*/∂*x*), *u* is the streamwise velocity in the direction of the fluid flow, *v* is the velocity in the direction normal to *u*, *ν* is the constant kinematic viscosity, and *U*(*x*) is the velocity at the edge of the boundary-layer which obeys the power-law relation *U*(*x*) = *ax* ^*m*^, (*x* > 0), where *a* is the mean stream velocity and *m* is a constant. The relevant boundary conditions for fixed plate are

(3) $$\begin{array}{c} y=0:u=0,\;v=0;\;u⟶a\;\;\text{as}\;\;y⟶\infty . \end{array}$$

A stream function *ψ*(*x*, *y*) is introduced such that

(4) $$\begin{array}{c} u=\frac{\partial \psi }{\partial y},\;\;v=-\frac{\partial \psi }{\partial x}. \end{array}$$

Equation (2) of continuity is satisfied identically. The momentum equation (1) becomes

(5) $$\begin{array}{c} \frac{\partial \psi }{\partial y}\frac{\partial^{2}\psi }{\partial x\partial y}-\frac{\partial \psi }{\partial x}\frac{\partial^{2}\psi }{\partial y^{2}}=U\frac{\partial U}{\partial x}+\nu \frac{\partial^{3}\psi }{\partial y^{3}}. \end{array}$$

Integrating (5) and using similarity variable yield

(6) $$\begin{array}{c} \psi =x^{\left(1+m\right)/2}\sqrt{\frac{2\nu a}{1+m}}F\left(\eta \right),\\ \eta =\frac{y}{x^{(1-m)/2}}\sqrt{\frac{\left(1+m\right)a}{2\nu }}. \end{array}$$

Substituting (6) into (5) gives the equation of Falkner-Skan in the form

(7) $$\begin{array}{c} F^{\prime \prime \prime }\left(\eta \right)+F\left(\eta \right)F^{\prime \prime }\left(\eta \right)+\beta \left(1-F^{\prime }{\left(\eta \right)}^{2}\right)=0 \end{array}$$

with the initial and boundary conditions

(8) $$\begin{array}{c} F\left(0\right)=0,\;F^{\prime }\left(0\right)=0,\;F^{\prime }\left(\infty \right)=0, \end{array}$$

where *β* = 2*m*/(*m* + 1) is a measure of the pressure gradient and prime denotes derivative with respect to  *η*.

## 3. Fundamentals of the OHAM

In what follows, we consider nonlinear differential equation

(9) $$\begin{array}{c} L\left(F\left(\eta \right)\right)+N\left(F\left(\eta \right)\right)=0 \end{array}$$

with boundary/initial condition

(10) $$\begin{array}{c} B\left(F,F^{\prime },F^{\prime \prime },\ldots \right)=0. \end{array}$$

In (9),  *L*  is a linear operator and  *N*  is a nonlinear operator. In (10),  *B*  is a boundary operator.

According to the basic ideas of OHAM [26–28], one constructs a family of equations

(11) (1−p)L(F(η,p))  =H(η,p)[L(F(η,p))+N(F(η,p))].

The boundary condition is

(12) $$\begin{array}{c} B\left(F\left(\eta ,p\right),\frac{\partial F\left(\eta ,p\right)}{\partial \eta },\frac{\partial^{2}F\left(\eta ,p\right)}{\partial \eta^{2}},\ldots \right)=0, \end{array}$$

where  *η* ∈ *R*,  *F*(*η*, *p*) is an unknown function, *p* ∈ [0, 1] is an embedding parameter, and *H*(*η*, *p*) is an auxiliary function such that *H*(*η*, 0) = 0 and *H*(*η*, *p*) ≠ 0 for *p* ≠ 0. When *p* increases from 0 to 1, the solution  *F*(*η*, *p*), changes from initial approximation *F*_0_(*η*) to the solution *F*(*η*). For *p* = 0 and *p* = 1 it holds that, respectively,

(13) $$\begin{array}{c} F\left(\eta ,0\right)=F_{0}\left(\eta \right),\;\;F\left(\eta ,1\right)=F\left(\eta \right). \end{array}$$

Expanding *F*(*η*, *p*) in series with respect to the parameter  *p*, one has

(14) $$\begin{array}{c} F\left(\eta ,p\right)=F_{0}\left(\eta \right)+pF_{1}\left(\eta \right)+p^{2}F_{2}\left(\eta \right)+\cdots . \end{array}$$

The series (14) contains the auxiliary function  *H*(*η*, *p*)  which determines their convergence region. For the auxiliary function  *H*(*η*, *p*)  we propose that

(15) $$\begin{array}{c} H\left(\eta ,p\right)=pH_{1}\left(\eta ,C_{j}\right)+p^{2}H_{2}\left(\eta ,C_{j}\right)+\cdots , \end{array}$$

where *H*_*i*_(*η*, *C*_*j*_) and *i* = 1, 2,… are functions of variable *η* and of a number of unknown parameters *C*_*j*_, *j* = 1, 2,…*q*. In this paper we consider the *m*th-order approximation in the form

(16) $$\begin{array}{c} \bar{F}\left(\eta \right)\approx F_{0}\left(\eta \right)+F_{1}\left(\eta \right)+\cdots +F_{m}\left(\eta \right). \end{array}$$

Inserting (14) into (11) we obtain

(17) L(F(η,p))+N(F(η,p))  =N0(F0(η))+pN1(F0(η),F1(η))   +p2N2(F0(η),F1(η),F2(η))+⋯,

where *N*_*i*_(*F*_0_, *F*_1_,…, *F*_*i*_) is the coefficient of *p* ^*i*^ in the expansion of *L*(*F*) + *N*(*F*) about the embedding parameter *p*.

Substituting (14) and (15) into (11) and equating the coefficients of like powers of *p*, we obtain the following linear equations:

(18) $$\begin{array}{c} L\left(F_{0}\left(\eta \right)\right)=0,\;\;B\left(F_{0}\left(\eta \right),F_{0}^{\prime }\left(\eta \right),F_{0}^{\prime \prime }\left(\eta \right),\ldots \right)=0, \end{array}$$

(19) L(Fi(η))−L(Fi−1(η))−∑j=1iHjNi−j(F0,F1,…,Fi−j)=0B(Fi,Fi′,Fi′′,…)=0, i=1,2,…,m−1,

(20) L(Fm(η))−L(Fm−1(η))−∑j=1m−1HjNm−1−j−HmN0=0B(Fm,Fm′,Fm′′,…)=0.

At this moment, the  *m*th-order approximate solution (16) depends on the functions *H*_1_(*η*, *C*_*i*_), *H*_2_(*η*, *C*_*i*_),…, *H*_*m*_(*η*, *C*_*i*_). The parameters *C*_1_, *C*_2_, …, *C*_*q*_ which appear in the expression of *H*_*i*_, *i* = 1, 2,…, *m* can be identified optimally via various methodologies such as the least square method, the Galerkin method, and the collocation method. The parameters *C*_1_, *C*_2_, …, *C*_*q*_ can be determined, for example, if we substitute (16) into (9), such that the residual becomes

(21) $$\begin{array}{c} R\left(\eta ,C_{i}\right)=L\left(\bar{F}\left(\eta ,C_{i}\right)\right)+N\left(\bar{F}\left(\eta ,C_{i}\right)\right),\;i=1,2,\ldots ,q. \end{array}$$

If *a* and *b* are two values from the domain of the problem and *η*_*i*_ ∈ (*a*, *b*), *i* = 1, 2,…, *q*, then the residual (21) must vanish

(22) R(η1,Ci)=R(η2,Ci)=⋯=R(ηq,Ci)=0,i=1,2,…,q

with* q*—the number of parameters *C*_*i*_ which appear in the expression of the functions *H*_*j*_(*η*),  *j* = 1, 2,…, *m*.

We remark that our procedure contains the auxiliary functions *H*_1_, *H*_2_,… which provides us with a simple but rigorous way to adjust and control convergence of the solution. It must be underlined that it is very important to properly choose the functions *H*_1_, *H*_2_,…, *H*_*m*_ which appear in the *m*th-order approximation (16). With these parameters known, the approximate solution is well determined. The parameters *C*_1_, *C*_2_,… are, namely, convergence-control parameters.

## 4. Application of OHAM to Falkner-Skan Equation

To use the basic ideas of the proposed method, we choose the linear operator

(23) $$\begin{array}{c} L\left(F\left(\eta ,p\right)\right)=\frac{\partial^{3}F\left(\eta ,p\right)}{\partial \eta^{3}}+K\frac{\partial^{2}F\left(\eta ,p\right)}{\partial \eta^{2}}, \end{array}$$

where  *K*  is the unknown parameter at this moment.

The nonlinear operator is

(24) N(F(η,p))=F(η,p)∂2F(η,p)∂η2−K∂2F(η,p)∂η2 +β[1−(∂F(η,p)∂η)2].

The boundary conditions are

(25) $$\begin{array}{c} F\left(0,p\right)=0,\;\;\frac{\partial F\left(0,p\right)}{\partial \eta }=0,\;\;\frac{\partial F\left(\infty ,p\right)}{\partial \eta }=1. \end{array}$$

Equation (18) can be written in the form

(26) $$\begin{array}{c} F_{0}^{\prime \prime \prime }\left(\eta \right)+KF_{0}^{\prime \prime }\left(\eta \right)=0,\\ F_{0}\left(0\right)=0,\;F_{0}^{\prime }\left(0\right)=0,\;F_{0}^{\prime }\left(\infty \right)=1 \end{array}$$

and has the solution

(27) $$\begin{array}{c} F_{0}\left(\eta \right)=\eta +\frac{e^{-K\eta }-1}{K}. \end{array}$$

From (17), (23), and (24) one obtain the expression

(28) $$\begin{array}{c} N_{0}\left(\eta \right)=-KF_{0}^{\prime \prime }\left(\eta \right)+F_{0}\left(\eta \right)F_{0}^{\prime \prime }\left(\eta \right)+\beta \left[1-F_{0}^{\prime }{\left(\eta \right)}^{2}\right]. \end{array}$$

Substituting (27) into (28), we obtain

(29) $$\begin{array}{c} N_{0}\left(\eta \right)=\left(K\eta +2\beta -1-K\right)e^{-K\eta }+\left(1-\beta \right)e^{-2K\eta }. \end{array}$$

If we consider the first-order approximate solution (*m* = 1), (16) becomes

(30) $$\begin{array}{c} \bar{F}\left(\eta \right)=F_{0}\left(\eta \right)+F_{1}\left(\eta \right), \end{array}$$

where *F*_1_(*η*) is obtained from (20):

(31) $$\begin{array}{c} F_{1}^{\prime \prime \prime }\left(\eta \right)+KF_{1}^{\prime \prime }\left(\eta \right)-\left(F_{0}^{\prime \prime \prime }\left(\eta \right)+KF_{0}^{\prime \prime }\left(\eta \right)\right)=H_{1}\left(\eta ,C_{i}\right)N_{0}\left(\eta \right). \end{array}$$

Substituting (27) and (29) into (31) we obtain the equation

(32) F1′′′(η)+KF1′′(η) =H1(η,Ci)[(Kη+2β−1−K)e−Kη+(1−β)e−2Kη],F1(0)=0, F1′(0)=0, F1′(∞)=0.

There are many possibilities to choose the function *H*_1_(*η*, *C*_*i*_) which appears into (32). The convergence of the solution *F*_1_(*η*) and consequently the convergence of the approximate solution $\bar{F}(\eta )$ given by (30) depend on the auxiliary function *H*_1_(*η*, *C*_*i*_). Basically, the shape of *H*_1_(*η*, *C*_*i*_) should follow the term appearing in (29) which is the product of polynomial and exponential functions. In general, we try to choose the function *H*_1_(*η*, *C*_*i*_) so that the product *H*_1_(*η*, *C*_*i*_)*N*_0_(*η*) from (31) and *N*_0_(*η*) would be of the same form. In our paper, for example, we can consider only the possibilities

(33) H1(η,Ci)=C1+C2η+C3η2+⋯+Cqηq−1,H1(η,Ci)=C1+C2η+C3e−Kη+C4e−2Kη,H1(η,Ci)=C1+C2η+C3η2+(C4+C5η)e−Kη

and so on, where *C*_1_, *C*_2_,… are unknown parameters. In the following we have four cases.

### 4.1. Case 1

If the auxiliary convergence-control function *H*_1_(*η*, *C*_*i*_) has the form

(34) H1(η,Ci)=C1+C2η+C3η2+C4η3 +(C5+C6η+C7η2)e−Kη+C8e−2Kη

then (32) can be written as

(35) F1′′′(η)+KF1′′(η) ={(2β−1−K2)C1+[(2β−1−K2)C2+KC1]η    +[(2β−1−K2)C3+KC2]η2    +[(2β−1−K2)C4+KC3]η3+KC4η4}e−Kη   +{(1−β)C1+(2β−1−K2)C5      +[(1−β)C2+KC5+(2β−1−K2)C6]η      +[(1−β)C3+KC6+(2β−1−K2)C7]η2      +[(1−β)C4+KC7]η3}e−2Kη +{(1−β)C5+(2β−1−K2)C8    +[(1−β)C6+KC8]η+(1−β)C7η2}e−3Kη +(1−β)C8e−4Kη.

Finally, using (27) and solving (35), we determine the first-order approximate solution given by (30) in the form

(36) F¯(η)=η−1K+4K2−7β−54K3C1+8K2−15β−174K4C2 +48K2−93β−1478K5C3+96K2−189β−3874K6C4 +9K2−14β−436K3C5+54K2−92β−43216K4C6 +81K2−146β−97216K5C7+48K2−69β−11432K3C8 +[C45K·η5+(C34K+2β+7−K24K2C4)·η4    +(C23K+2β+5−2K23K2C3      +4β+10−2K2K3C4)·η3    +(C12K+2β+3−K22K2C2+4β+7−2K2K3C3    +18β+39−9K2K4C4)·η2    +(2β+1−K2K2C1+4β+4−2K2K3C2      +12β+18−6K2K4C3      +48β+96−24K2K5C4)·η    +1K+3β+3−2K22K3C1+13β+19−8K24K4C2    +41β+79−24K24K5C3+339β+813−192K28K6C4    +10β+5−6K212K3C5+49β+41−27K236K4C6    +718β+875−378K2216K5C7    +9β+2−6K236K3C8]·e−Kη +[(β−14K3C4−14K2C7)·η3    +(β−14K3C3+3β−32K4C4−14K2C6       +K2−5−2β4K3C7)·η2    +(β−14K3C2+β−1K4C3+33β−338K5C4−14K2C5    +K2−2β−34K3C6+8K2−16β−258K4C7)·η    +β−14K3C1+β−12K4C2+11β−118K5C3    +39β−398K6C4+K2−2β−14K3C5    +4K2−8β−78K4C6+11K2−22β−288K5C7]·e−2Kη +[β−118K3C7η2    +(β−118K3C6+7β−754K4C7−118K2C8)·η    +β−118K3C5+7β−7108K4C6+11β−11108K5C7    +6K2−1−12β108K3C8]·e−3Kη+β−148K3C8e−4Kη.

### 4.2. Case 2

The auxiliary function *H*_1_(*η*, *C*_*i*_) has the form

(37) H1(η,Ci)=C1+C2η+C3η2+C4η3+C5η4 +(C6+C7η+C8η2)e−Kη.

In this case, (32) becomes

(38) F1′′′(η)+KF1′′(η) ={(2β−1−K2)C1+[(2β−1−K2)C2+KC1]η   +[(2β−1−K2)C3+KC2]η2     +[(2β−1−K2)C4+KC3]η3   +[(2β−1−K2)C5+KC4]η4+KC5η5}e−Kη  +{(1−β)C1+(2β−1−K2)C6    +[(1−β)C2+KC6+(2β−1−K2)C7]η    +[(1−β)C3+KC7+(2β−1−K2)C8]η2    +[(1−β)C4+KC8]η3+(1−β)C5η4}e−2Kη  +[(1−β)C6+(1−β)C7η+(1−β)C8η2].

The first-order approximate solution (30) in this case is obtained from (38) and (27) and can be written as

(39) F¯(η) =η−1K+4K2−7β−54K3C1+8K2−15β−174K4C2  +48K2−93β−1478K5C3+96K2−189β−3874K6C4  +960K2−1935β−47858K7C5+9K2−14β−436K3C6  +54K2−92β−43216K4C7+81K2−146β−97216K5C8  +[C56Kη6+(C45K+2β+9−K29K2C5)·η5   +(C34K+2β+7−K24K2C4+4β+13−2K2K3C5)·η4   +(C23K+2β+5−K23K2C3+4β+10−2K2K3C4      +24β+68−12K2K4C5)·η3   +(C12K+2β+3−K22K2C2+4β+7−2K2K3C3      +18β+39−9K2K4C4+96β+252−48K2K5C5)·η2   +(2β+1−K2K2C1+4β+4−2K2K3C2      +12β+18−6K2K4C3+48β+96−24K2K5C4      +240β+600−120K2K6C5)·η   +1K+3β+3−2K22K3C1+13β+19−8K24K4C2   +41β+79−24K24K5C3+339β+813−192K28K6C4   +1053β+2307−480K24K7C5+10β+5−6K212K3C6   +49β+41−27K236K4C7   +718β+875−378K2216K5C8]·e−Kη  +[β−14K3C5η4+(β−14K3C4−2β−2K4C5−14K2C8)·η3     +(β−14K3C3+3β−32K4C4+33β−334K5C5−14K2C7        +2β−7−K24K3C8)·η2     +(β−14K3C2+β−1K4C3+33β−338K5C4          +39−39β2K6C5−14K2C6+K2−2β−34K3C7          +8K2−16β−258K4C8)·η+β−14K3C1+β−12K4C2     +11β−118K5C3+39β−398K6C4+171β−1718K7C5     +K2−2β−14K3C6+4K2−8β−78K4C7     +11K2−22β−288K5C8]·e−2Kη  +[β−118K3C8η2+(β−118K3C7+7β−754K4C8)·η     +β−118K3C6+7β−7108K4C7+11β−11108K5C8]·e−3Kη.

### 4.3. Case 3

If the auxiliary function *H*_1_(*η*, *C*_*i*_) has the form

(40) H1(η,Ci)=C1+C2η+C3η2+C4η3+(C5+C6η+C7η2+C9η3)e−Kη+C8e−2Kη

then the first-order approximate solution equation (30) has the form

(41) F¯¯(η)=F¯(η)+C9[−6804K2+793+908β648K6   +1215K2+3872+2365β216K6e−Kη   +(−14K2η4+K2−7−2β4K3η3     +6K2−27−12β4K4η2     +33K2−123−66β8K5η     +39K2−132−78β8K6)e−2Kη   +(β−118K3η3+7β−736K4η2     +11β−1136K5η+131β−131648K6)e−3Kη],

where $\bar{F}(\eta )$ is given by (36).

### 4.4. Case 4

In the last case, we consider

(42) H1(η,Ci)=C1+C2η+C3η2+C4η3+C5η4 +(C6+C7η+C8η2+C9η3)e−Kη

such that the first-order approximate solution equation (30) becomes

(43) F¯¯(η)=F¯(η) + C9[−6804K2+793+908β648K6       +(−14K2η4+1215K2+3872+2365β216K6e−Kη       +K2−7−2β4K3η3+6K2−27−12β4K4η2       +33K2−123−66β8K5η       +39K2−132−78β8K6)e−2Kη       +(β−118K3η3+7β−736K4η2+11β−1136K5η     +131β−131648K6)e−3Kη],

where $\bar{F}(\eta )$ is given by (39).

## 5. Numerical Examples

In order to prove the accuracy of the obtained results, we will determine the convergence-control parameters *C*_*i*_ which appear in (36), (39), (41), and (43) by means of Galerkin method. Let *R*(*η*, *C*_*i*_) be the residual within the approximate solution $\bar{F}(\eta )$ (or $\bar{\bar{F}}(\eta )$) given by (36), (39), (41), and (43) which satisfies (7):

(44) R(η,Ci)=F¯′′′(η,Ci)+F¯(η,Ci)F¯′′(η,Ci) +β(1−F¯′2(η,Ci)).

Since *R*(*η*, *C*_*i*_) contains the parameters *C*_*i*_, *i* = 1,2,…. The parameters can be determined from the conditions

(45) Ji(Cj)=∫0∞R(η,Cj)fi(η)dη=0,i=1,2,…,q, j=1,2,…,q,

where *f*_*i*_ are linear independent functions, taken as weighting functions. Equations (36) and (39) contain nine unknown parameters: *K* and *C*_*i*_, *i* = 1,2,…, 8, and therefore we consider the following nine weighting functions (*q* = 9):

(46) $$\begin{array}{c} f_{1}=e^{-K\eta },\;\;f_{2}=\eta e^{-K\eta },\;\;f_{3}=\eta^{2}e^{-K\eta },\\ f_{4}=\eta^{3}e^{-K\eta },\;\;f_{5}=\eta^{4}e^{-K\eta },\;\;f_{6}=\eta^{5}e^{-K\eta },\\ f_{7}=\eta e^{-2K\eta },\;\;f_{8}=\eta^{2}e^{-2K\eta },\;\;f_{9}=\eta^{3}e^{-2K\eta }. \end{array}$$

For (41) and (43) which contain the unknown parameters: *K*, *α*, and *C*_*i*_, *i* = 1, 2,…, 9  , we consider weighting functions (*q* = 11)

(47) $$\begin{array}{c} f_{1}=e^{-K\eta },\;\;f_{2}=\eta e^{-K\eta },\;\;f_{3}=\eta^{2}e^{-K\eta },\\ f_{4}=\eta^{3}e^{-K\eta },\;\;f_{5}=\eta^{4}e^{-K\eta },\;\;\\ f_{6}=\eta^{4}e^{-2K\eta }+\alpha \eta e^{-4K\eta },\\ f_{7}=\eta e^{-2K\eta },\;\;f_{8}=\eta^{2}e^{-2K\eta },\;\;f_{9}=\eta^{3}e^{-2K\eta },\\ f_{10}=\eta^{5}e^{-K\eta }+\eta e^{-3K\eta },\;\;f_{11}=\eta^{3}e^{-4K\eta }+\eta^{7}e^{-2K\eta }. \end{array}$$

In this way, the convergence-control parameters *C*_*i*_, *i* = 1,2,… are optimally determined and the first-order approximate solutions are known for different values of the known parameter *β*.

In what follows, we illustrate the accuracy of the OHAM comparing previously obtained approximate solutions with the numerical integration results computed by means of the shooting method combined with fourth-order Runge-Kutta method using Wolfram Mathematica 6.0 software. Also we will show that the error of the solutions decreases when the number of terms in the auxiliary convergence-control function *H*_1_ increases. For some values of the parameter *β*, we will determine the approximate solutions given by (36), (39), (41), and (43) and with the unknown parameters *α*, *K*, and *C*_*i*_ obtained from the system given by (45).

**Example 1**

In the first case we consider that *β* = 1/2.

(a) For (36) and from the system (45), following the procedure described above the convergence-control parameters are obtained

(48) $$\begin{array}{c} C_{1}=-0.0974633576,\;\;C_{2}=0.3334342895,\\ C_{3}=-0.0846971328,\;\;C_{4}=0.0054445300,\\ C_{5}=10.8640621331,\;\;C_{6}=-8.9072774043,\\ C_{7}=0.7917175772,\;\;C_{8}=0.4377125759,\\ K=0.9345058664 \end{array}$$

and consequently the first-order approximate solution (36) can be written in the form

(49) F¯(η) =−0.8095502989+η  +(−2.2237146927+0.2852293525η   +0.0785389555η2+0.0216006979η3   +0.0115505513η4+0.0011652211η5)e−0.9345058664η  +(3.0954492754+2.0345244657η   +0.8905286290η2−0.1261379297η3)e−1.8690117328η  +(−0.0565973734+0.2080470361η   −0.0269476991η2)e−2.8035175992η  −0.0055869102·e−3.7380234657η.

In Tables 1 and 2 we present a comparison between the first-order approximate solution given by (49) and velocity obtained from (49), respectively, with numerical results for some values of variable *η* and the corresponding relative errors.

(b) From (39), obtained by means of the auxiliary convergence-control function *H*_1_ given by (37), we obtain the following results for the parameters:

(50) $$\begin{array}{c} C_{1}=15.3365053132,\;\;C_{2}=-20.7743165892,\\ C_{3}=9.3076203696,\;\;C_{4}=-2.0941285422,\\ C_{5}=0.2277189607,\;\;C_{6}=-5.2584096507,\\ C_{7}=-45.5148999446,\;\;C_{8}=8.3240015527,\\ K=2.1171968259. \end{array}$$

The first-order approximate solution (39) becomes

(51) F¯(η)=−0.8071635483+η   +(2.8068611339−3.4953295790η    +4.4303812597η2−2.5930631506η3    +0.8330363534η4−0.1666768365η5    +0.0179261368η6)e−2.1171968259η   +(−2.0785335331+0.0310432086η    +0.2175675516η2−0.4253321356η3    −0.0029993386η4)e−4.2343936518η   +(0.0788359475+0.1063683100η    −0.0243638517η2)e−6.3515904777η.

In Tables 3 and 4 we present some values of stream function (51) and velocity obtained from (51), respectively, for different values of *η* and the corresponding relative errors.

(c) For (41), which depends on the auxiliary convergence-control function *H*_1_ given by (40), we obtain

(52) $$\begin{array}{c} C_{1}=17.1086190380,\;\;C_{2}=-6.1886479153,\\ C_{3}=0.7626899878,\;\;C_{4}=-0.0317286051,\\ C_{5}=-63.8867694459,\;\;C_{6}=0.7750251041,\\ C_{7}=-7.4945476964,\;\;C_{8}=-9.8195426109,\\ C_{9}=-0.4164447206,\;\;K=1.1269038309,\\ \alpha =0.8746910670. \end{array}$$

Therefore, the first-order approximate solution for stream function is

(53) F¯¯(η)=−2.4537934149+η   +(−18.9229176282+1.0563974022η     +3.6621795605η2−1.0933800148η3     +0.1271627184η4     +0.0056311114η5)e−1.1269038309η   +(19.9100776837+25.8158411889η     +9.3137451766η2+1.9358858129η3)   ×e−2.25380766196η   +(1.3951574877+0.7157493617η    +0.1454729886η2)e−3.3807114929η   +0.0714758716e−4.5076153239η.

In Tables 5 and 6 we present some values of stream function (53) and velocity obtained from (53), respectively, for different values of variable *η* and the corresponding relative errors.

Comparing the results presented in Table 1 with the results presented in Table 5 and, on the other hand, the results presented in Table 2 with the results presented in Table 6, respectively, it is clear that the analytical solutions obtained by our procedure prove to be more accurate along with an increased number of terms in the auxiliary convergence-control function *H*_1_.

(d) If we consider (43) depending on the auxiliary convergence-control function *H*_1_ given by (42) then from system (45) we obtain the following results:

(54) $$\begin{array}{c} C_{1}=28.8801485140,\;\;C_{2}=-30.7469629234,\\ C_{3}=10.1656115824,\;\;C_{4}=-1.3058736876,\\ C_{5}=0.0629713196,\;\;C_{6}=-11.2295919004,\\ C_{7}=62.3525565579,\;\;C_{8}=-18.6274524032,\\ C_{9}=13.7997786872,\;\;K=1.9932224781,\\ \alpha =2.7977542979. \end{array}$$

The first-order approximate solution for stream function (43) becomes

(55) F¯¯(η)=−0.8042841228+η +(5.055972832−7.4607384673η    +7.6988739549η2−3.9526112515η3    +1.0002001924η4−0.1204170249η5    +0.0052654533η6)e−1.9932224781η +(−4.126293156−0.2733114331η      −0.151609344η2−0.5576699112η3      −0.8693555337η4)e−3.9864449562η +(−0.0807202735−0.2092400883η    −0.0196584681η2−0.0484063456η3) ×e−5.9796674343η.

In Tables 7 and 8 we present a comparison between the first-order approximate solution given by (55) and velocity obtained from (55), respectively, with numerical results for some values of variable *η* and the corresponding relative errors.

If we compare the results presented in Tables 3 and 7 and then the results presented in Tables 4 and 8, respectively, we can arrive at conclusion that the analytical results obtained by OHAM are more accurate along with an increased number of terms in the auxiliary convergence-control function *H*_1_. It is important to establish the value of the shear-stress profile *F*′′(0). In Table 9 we present a comparison between the values of ${\bar{F}}^{\prime \prime }(0)$ obtained using OHAM from (49), (51), (53), and (55) and numerical results for *β* = 1/2. Our results are in very good agreement with the numerical results.

**Example 2**

In this second case we suppose that *β* = 1.

(a) For (36), from the system (45) we obtained the values of the convergence-control parameters:

(56) $$\begin{array}{c} C_{1}=5.9913279914,\;\;C_{2}=-6.6828413743,\\ C_{3}=2.0729320860,\;\;C_{4}=-0.1950415110,\\ C_{5}=18.0871429890,\;\;C_{6}=-84.4868066767,\\ C_{7}=17.6775180449,\;\;C_{8}=-0.3871636300,\\ K=2.0064973399. \end{array}$$

For these values of the parameters, from (36) we obtained the first-order approximate solution in the form

(57) F¯(η)=−0.6476658539+η +(1.1858804994−1.0205705493η  +1.1912223638η2−0.7433934009η3  +0.1980363504η4−0.0194409937η5) ×e−2.0064973399η +(−0.5332638964+0.2249079388η  +0.3083821306η2+0.0240412688η3) ×e−4.0129946798η +(−0.004950749+0.0053425041η)e−6.0194920197η.

In Tables 10 and 11 we present some values of stream function given by (57) and velocity obtained from (57), respectively, for different values of variable *η*. Also the corresponding relative errors are given in these cases.

(b) For (39), the system (45) has the solutions

(58) $$\begin{array}{c} C_{1}=25.6413506662,\;\;C_{2}=-27.7257520445,\\ C_{3}=10.2440262047,\;\;C_{4}=-1.6311566772,\\ C_{5}=0.1133872279,\;\;C_{6}=-37.8620215531,\\ C_{7}=4.5810479839,\;\;C_{8}=-9.7143709526,\\ K=2.0340821793. \end{array}$$

The first-order approximate solution can be written as

(59) F¯(η)=−0.6482098275+η +(1.7192094539−4.5907375859η  +5.3333876914η2−3.0101935973η3  +0.8973516349η4−0.1394863598η5  +0.0092906134η6)e−2.0340821793η +(−1.0709996263+2.7307483905η  +2.3599924746η2+0.5869724289η3) ×e−4.0681643586η.

In Tables 12 and 13 we present some values of stream function (59) and velocity obtained from (59), respectively, for different values of variable *η* and the corresponding relative errors.

(c) For (41) with the auxiliary convergence-control function *H*_1_ given by (40) we have

(60) $$\begin{array}{c} C_{1}=15.1069993472,\;\;C_{2}=-6.1116892850,\\ C_{3}=0.8419581636,\;\;C_{4}=-0.0391594463,\\ C_{5}=-348.1410130441,\;\;C_{6}=354.6124244346,\\ C_{7}=-78.2878044901,\;\;C_{8}=-3.8910409852,\\ C_{9}=-4.3179402181,\;\;K=1.3940527605,\\ \alpha =1.2080121437. \end{array}$$

The first-order approximate solution for stream function (41) becomes

(61) F¯¯(η)=−0.6477776791+η +(−18.001344737+2.2989361198η  +2.4130328127η2−0.8773035418η3  +0.1154431274η4−0.0056180723η5) ×e−1.3940527605η +(18.631306321+23.515563981η  +12.357602161η2+3.3122912941η3  +0.5554669357η4)e−2.7881055211η +(0.0178160913+0.1112333145η)e−4.1821582817η.

In Tables 14 and 15 we present some values of stream function (61) and velocity obtained from (61), respectively, for different values of variable *η* and the corresponding relative errors.

(d) If we have in view (43), with the auxiliary convergence-control function *H*_1_ given by (42), then we obtain

(62) $$\begin{array}{c} C_{1}=2.5755597425,\;\;C_{2}=-0.5965183787,\\ C_{3}=-0.0172692621,\;\;C_{4}=0.0136562951,\\ C_{5}=-0.0010079176,\;\;C_{6}=-5.9530458601,\\ C_{7}=-1.9930045747,\;\;C_{8}=-1.9931630292,\\ C_{9}=0.0743455822,\;\;K=1.1143724942,\\ \alpha =0.2855970092 \end{array}$$

and therefore first-order approximate solution (43) is given by

(63) F¯¯(η)=−0.6475361092+η +(−7.0772773941+1.5599095672η  +0.3833481708η2−0.141867195η3  +0.0068820136η4+0.0015709234η5  −0.0001507451η6)e−1.1143724942η +(7.7248135031+6.7700065534η  +2.2685016445η2+0.2970574871η3  −0.014966987η4)e−2.2287449884η.

In Tables 16 and 17 we present a comparison between the first-order approximate solution given by (63) and velocity obtained from (63), respectively, with numerical results and the corresponding relative errors.

If we compare the results presented in Tables 10 and 14 and then the results presented in Tables 11 and 15 we deduce that the analytical results obtained by OHAM are more accurate along with an increased number of terms in the auxiliary convergence-control function *H*_1_. The same conclusions are deduced if we compare the results presented in Tables 12 and 16 and then the results presented in Tables 13 and 17, respectively.

In Table 18 we present a comparison between the values of ${\bar{F}}^{\prime \prime }(0)$ obtained using OHAM from (57), (59), (61), and (63) and numerical integration results from *β* = 1. We can deduce that the results obtained by means of OHAM are nearly identical with those obtained through numerical integration.

**Example 3**

In the last case we consider *β* = 1.6.

(a) For (36), the values of the convergence-control parameters are obtained from the system (45):

(64) $$\begin{array}{c} C_{1}=-4.4749746430,\;\;C_{2}=2.6648959478,\\ C_{3}=-0.5277449747,\;\;C_{4}=0.0344313836,\\ C_{5}=0.9084258281,\;\;C_{6}=11.1816458907,\\ C_{7}=-0.5492649796,\;\;C_{8}=1.5695573134,\\ K=1.7389825314. \end{array}$$

The first-order approximate solution for stream function (36) can be written as

(65) F¯(η)=−0.5440478033+η +(1.6577374986−0.4175138596η  −0.4722933401η2+0.2774993818η3  −0.05544381588η4+0.0039599458η5) ×e−1.7389825314η +(−1.1629370587−1.5183251610η  −0.7578665325η2−0.1287736808η3) ×e−3.4779650629η +(0.0455165672+0.0373698159η  −0.0034815689η2)e−5.2169475944η +0.0037307962e−6.9559301258η.

In Tables 19 and 20 we present some values of the stream function given by (65) and velocity obtained from (65), respectively, for different values of variable *η*. Also the corresponding relative errors are given in these tables.

(b) For (39), the convergence-control parameters obtained from the system (45) have the values

(66) $$\begin{array}{c} C_{1}=12.1397319477,\;\;C_{2}=-14.7300094090,\\ C_{3}=6.0298078722,\;\;C_{4}=-1.0787489057,\\ C_{5}=0.0852147302,\;\;C_{6}=-14.8695758423,\\ C_{7}=-7.8344847253,\;\;C_{8}=-2.4044068709,\\ K=2.0881719331. \end{array}$$

The first-order approximate solution for stream function (39) becomes

(67) F¯(η)=−0.544241252+η +(0.398631080−1.040810138η    +1.69949761η2−1.248940652η3    +0.460677086η4−0.086297165η5    +0.00680138η6)e−2.088171933η +(0.219770025+1.364996288η    +1.025546039η2+0.114703592η3    +0.001403806η4)e−4.176343866η +(−0.0741598535−0.038516290η    −0.008802133η2)e−6.264515799η.

In Tables 21 and 22 we present some values of the stream function (67) and velocity obtained from (67), respectively, and the corresponding relative errors in comparison with the numerical results.

(c) For (41), with the auxiliary convergence-control function *H*_1_ given by (40), the system (45) has the solutions

(68) $$\begin{array}{c} C_{1}=3.7343092835,\;\;C_{2}=-1.6919201004,\\ C_{3}=0.2585003215,\;\;C_{4}=-0.0131947678,\\ C_{5}=26.9486507241,\;\;C_{6}=-47.5028568351,\\ C_{7}=5.3389432018,\;\;C_{8}=-8.7586264382,\\ C_{9}=0.2924008927,\;\;K=1.4882721635,\\ \alpha =1974.2364175690. \end{array}$$

The first-order approximate solution for stream function (41) can be written as

(69) F¯¯(η)=−0.544001931+η +(−4.354906951+1.3261535367η  +0.3072449473η2+0.1940259496η3  +0.0315309032η4−0.0017731659η5) ×e−1.4882721635η +(4.8182102767+6.0137273891η  +2.4451256821η2+0.8109067414η3  −0.0330030433η4)e−2.976544327η +(0.1139109044−0.168675728η  +0.060940104η2+0.0029567211η3) ×e−4.4648164905η−0.033212299e−5.9530886541η.

In Tables 23 and 24 we present some values of stream function (69) and velocity obtained from (69), respectively, for different values of variable *η* and the corresponding relative errors.

(d) If we have in view (43) with the auxiliary convergence-control function *H*_1_ given by (42) then the convergence-control parameters obtained from system (45) are

(70) $$\begin{array}{c} C_{1}=-9.9309813061,\;\;C_{2}=6.8914081433,\\ C_{3}=-1.7496966452,\;\;C_{4}=0.2003197512,\\ C_{5}=-0.0103677381,\;\;C_{6}=10.1134115449,\\ C_{7}=7.0897290367,\;\;C_{8}=1.9899138244,\\ C_{9}=0.0159776712,\;\;K=1.5329158534,\\ \alpha =-0.9799303286. \end{array}$$

In this case, the first-order approximate solution for stream function (43) becomes

(71) F¯¯(η)=−0.5439219913+η +(4.650996506−2.0398248866η  −0.4858446135η2+0.550362856η3  −0.1603616877η4+0.0213068753η5  −0.0011272349η6)e−1.5329158534η +(−4.2651113968−4.2739140037η    −1.660992309η2−0.2098183934η3    −0.0021316125η4)e−3.0658317069η +(0.158036881+0.0939831378η  +0.0187520087η2+0.0001478554η3) ×e−4.5987475603η.

In Tables 25 and 26 we present some values of stream function given by (71) and velocity obtained from (71), respectively, for different values of variable *η*. Also the corresponding relative errors are given in these cases.

In Table 27 we present a comparison between the values of ${\bar{\bar{F}}}^{\prime \prime }(0)$ obtained through OHAM from (65), (67), (69), and (71) and numerical results for *β* = 1.6. We can observe that the analytical solutions obtained by OHAM are very accurate, being nearly identical with the numerical results.

**Figure 1:**
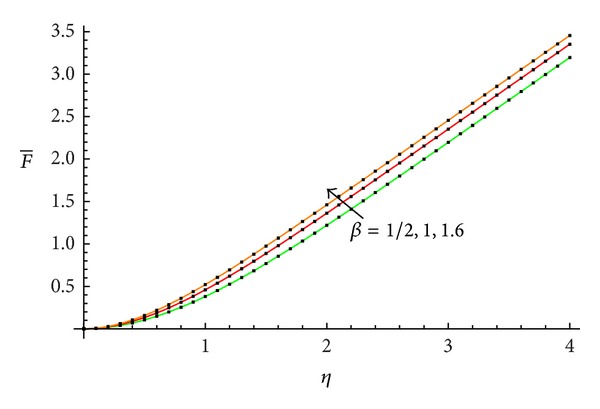
Comparison between the approximate solutions (49), (57), and (65) and numerical results with the auxiliary function equation (34) in the cases *β* = 1/2, 1, and 1.6, respectively: —numerical; …OHAM solution.

**Figure 2:**
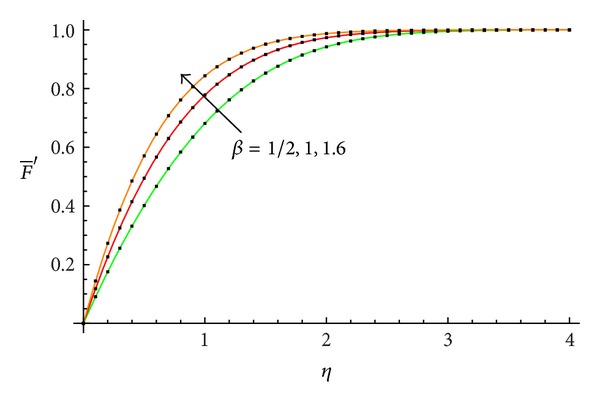
Comparison between the velocity profile obtained from (49), (57), and (65) and numerical results for *β* = 1/2, 1, and 1.6, respectively: —numerical; …OHAM solution.

**Figure 3:**
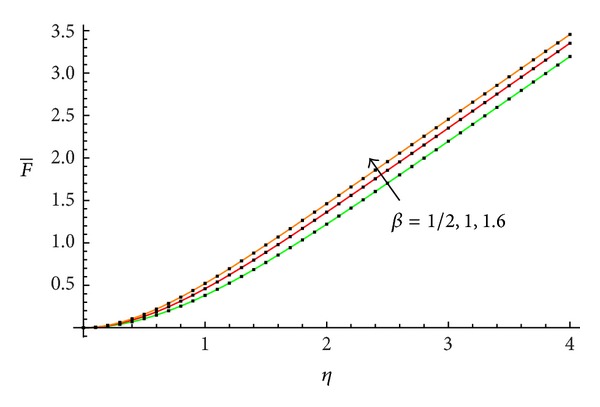
Comparison between the approximate solutions (51), (59), and (67) and numerical results with the auxiliary function equation (37) in the cases *β* = 1/2, 1, and 1.6, respectively: —numerical; …OHAM solution.

**Figure 4:**
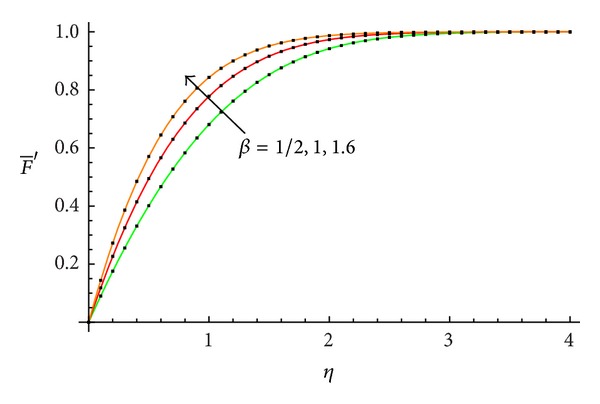
Comparison between the velocity profile obtained from (51), (59), and (67) and numerical results for *β* = 1/2, 1, and 1.6, respectively: —numerical; …OHAM solution.

**Figure 5:**
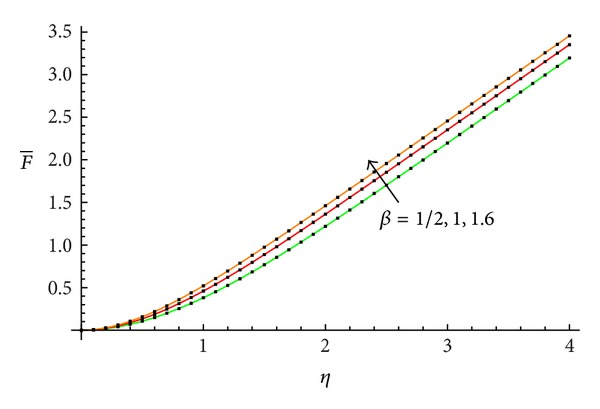
Comparison between the approximate solutions (53), (61), and (69) and numerical results with the auxiliary function equation (40) in the cases *β* = 1/2, 1, and 1.6, respectively: —numerical; …OHAM solution.

**Figure 6:**
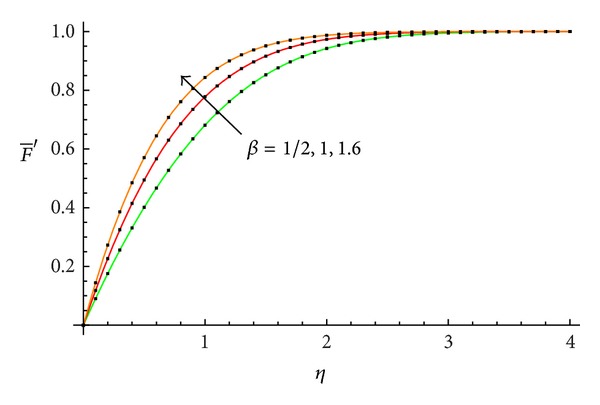
Comparison between the velocity profile obtained from (53), (61), and (69) and numerical results for *β* = 1/2, 1, and 1.6, respectively: —numerical; …OHAM solution.

**Figure 7:**
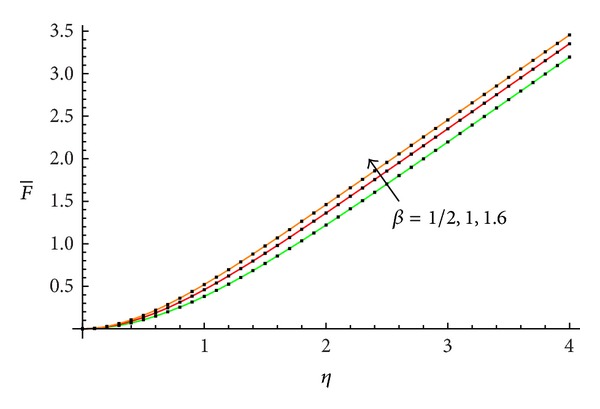
Comparison between the approximate solutions (55), (63), and (71) and numerical results with the auxiliary function equation (42) in the cases *β* = 1/2, 1, and 1.6, respectively: —numerical; …OHAM solution.

**Figure 8:**
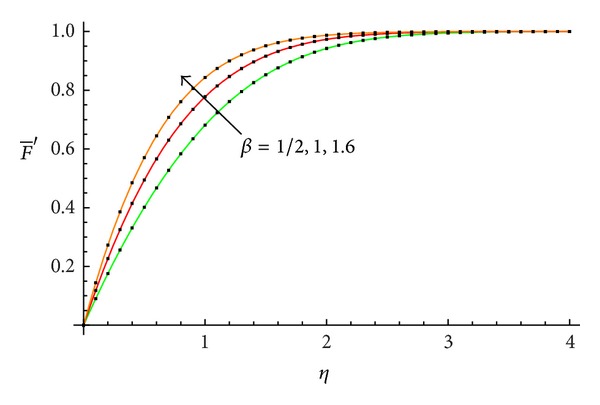
Comparison between the velocity profile obtained from (55), (63), and (71) and numerical results for *β* = 1/2, 1, and 1.6, respectively: —numerical; …OHAM solution.

The accuracy of the obtained results is verified graphically in Figures 1–8. The approximate solutions obtained by means of OHAM and with the auxiliary convergence-control functions *H*_1_ given by (34), (37), (40), and (42) are compared with numerical integration results for different values of *β* in Figures 1, 3, 5, and 7, respectively. On the other hand, the velocity profile obtained from the corresponding approximate solutions is compared with numerical integration results in Figures 2, 4, 6, and 8, respectively. In all cases presented in Figures 1–8, it is shown that the first-order approximate solutions and the velocity increase with an increase in the parameter *β*.

Just like the above mentioned cases, by comparing results presented in Tables 18 and 22 and then the results presented in Tables 19 and 23 we can write that the results obtained by OHAM are more accurate along with an increased number of terms in the auxiliary convergence-control function *H*_1_. Now, comparing the results from Tables 20 and 24 and then the results from Tables 21 and 25 the conclusions are the same.

## 6. Conclusions

In the present work we proposed an optimal homotopy approach to obtain approximate analytical solutions for nonlinear differential equation of Falkner-Skan. The validity of our procedure called optimal homotopy asymptotic method (OHAM) was demonstrated on some representative examples and very good agreement was found between the approximate analytic results and numerical simulation results. The proposed procedure is valid even if the nonlinear equation does not contain any small or large parameter. The basic equations governing an incompressible fluid subject to a pressure gradient are reduced to a nonlinear differential equation using similarity variables and are solved by means of OHAM. We examine quantitative effect of parameter *β* which is a measure of the pressure gradient and the relative errors of approximate solutions in comparison with numerical results.

To solve the equation of Falkner-Skan, we used OHAM, an approach proposed by Marinca and Herişanu [26–28]. For achieving a very accurate solution, OHAM ensured a very rapid convergence after only one iteration. Our procedure is a powerful approach for solving nonlinear problems without depending on small parameters.

The cornerstone of the validity and flexibility of our method is the choice of the linear operator *L* and the auxiliary convergence-control function *H*_1_. The convergence of the solutions depends on these auxiliary functions and implicitly on the presence of convergence-control parameters *α*, *K*, *C*_*i*_, and *i* = 1,2,….

Instead of an infinite series, the OHAM searches for only a few terms and does not need a recurrence formula. The parameters which appear in the composition of the auxiliary functions *H*_1_ and in the linear operator *L* are optimally identified via various methods by rigorously mathematical point of view. A large number of parameters in the auxiliary functions  *H*_1_  lead to a better accuracy of the results. In all cases presented in this paper, for different values of the parameter *β* we obtain an excellent agreement of the first-order approximate solutions.

Also, we obtain very good results by OHAM for different representative values of parameter *β* for shear-stress profiles ${\bar{F}}^{\prime \prime }(0)$ of the Falkner-Skan equation in comparison with the results obtained via numerical integration. It is worth mentioning that the proposed method is straightforward and concise and can be applied to other nonlinear problems.

It is interesting to remark that a large number of parameters *C*_*i*_ in the auxiliary convergence-control functions *H*_1_ lead to a better accuracy of the results (for the stream function and velocity). On the other hand, results obtained by OHAM are more accurate along with increased values of the parameter *β*.

## Figures and Tables

**Table 1:** Comparison between OHAM results given by (49) and numerical solutions for *β* = 1/2.

*η*	*F*_numerical_(*η*)	${\overset{-}{F}}_{\text{OHAM}}$(*η*) from (49)	Relative error = $\left|F_{\text{numerical}}(\eta )-{\overset{-}{F}}_{\text{OHAM}}(\eta )\right|$
0	−5.775724 · 10^−25^	5.551115 · 10^−16^	5.551115 · 10^−16^
4/5	0.2543480764	0.2543149422	0.0000331341
8/5	0.8550267840	0.8550621314	0.0000353473
12/5	1.6045273996	1.6043588322	0.0001685673
16/5	2.3963133788	2.3962404320	0.0000729467
4	3.1955002598	3.1953781616	0.0001220981
24/5	3.9954529746	3.9949610905	0.0004918840
28/5	4.7954513976	4.7946972370	0.0007541605
32/5	5.5954513676	5.5946526226	0.0007987450
36/5	6.3954513670	6.3946242000	0.0008271670
8	7.1954513667	7.19442747097	0.0010238957

**Table 2:** Comparison between OHAM results for velocity ${\bar{F}}^{\prime }(\eta )$ obtained from (49) and numerical results for *β* = 1/2.

*η*	*F*_numerical_′(*η*)	${\bar{F}}_{\text{OHAM}}^{\prime }(\eta )$ from (49)	Relative error = $\left|{F_{\text{numerical}}^{\prime }}_{}(\eta )-{\bar{F}}_{\text{OHAM}}^{\prime }(\eta )\right|$
0	1.852884 · 10^−21^	4.440892 · 10^−16^	4.440873 · 10^−16^
4/5	0.5833048177	0.5833149514	0.0000101337
8/5	0.8760975697	0.8759651454	0.0001324243
12/5	0.9760687561	0.9759519582	0.0001167979
16/5	0.9971920750	0.9973768978	0.0001848228
4	0.9998081986	0.9994693079	0.0003388907
24/5	0.9999925769	0.9995222975	0.0004702794
28/5	0.9999998398	0.9998331589	0.0001666808
32/5	0.9999999978	1.0000050268	5.028974 · 10^−6^
36/5	0.9999999995	0.9998832648	0.0001167346
8	0.9999999995	0.9996174949	0.0003825046

**Table 3:** Comparison between OHAM results given by (51) and numerical solutions for *β* = 1/2.

*η*	*F*_numerical_(*η*)	${\overset{-}{F}}_{\text{OHAM}}$(*η*) from (51)	Relative error = $\left|F_{\text{numerical}}(\eta )-{\overset{-}{F}}_{\text{OHAM}}(\eta )\right|$
0	−5.7757 · 10^−25^	−1.3322 · 10^−15^	1.3322 · 10^−15^
4/5	0.2543480764	0.2543333746	0.0000147017
8/5	0.8550267840	0.8549561291	0.0000706549
12/5	1.6045273996	1.6042714779	0.0002559216
16/5	2.3963133788	2.3958902142	0.0004231645
4	3.1955002598	3.1948843675	0.0006158922
24/5	3.9954529746	3.9943062991	0.0011466754
28/5	4.7954513976	4.7936944532	0.0017569444
32/5	5.5954513676	5.5932562778	0.0021950898
36/5	6.3954513670	6.3930163452	0.0024350218
8	7.1954513667	7.1929060945	0.0025452721

**Table 4:** Comparison between OHAM results for velocity ${\bar{F}}_{}^{\prime }(\eta )$ obtained from (51) and numerical results for *β* = 1/2.

*η*	*F*_numerical_′(*η*)	${\bar{F}}_{\text{OHAM}}^{\prime }(\eta )$ from (51)	Relative error = $\left|{F_{\text{numerical}}^{\prime }}_{}(\eta )-{\bar{F}}_{\text{OHAM}}^{\prime }(\eta )\right|$
0	1.852884 · 10^−21^	2.553512 · 10^−15^	2.553511 · 10^−15^
4/5	0.5833048177	0.5831981849	0.0001066327
8/5	0.8760975697	0.8760769428	0.0000206268
12/5	0.9760687561	0.9757219753	0.0003467808
16/5	0.9971920750	0.9970876920	0.0001043829
4	0.9998081986	0.9993456222	0.0004625764
24/5	0.9999925769	0.9992025737	0.0007900032
28/5	0.9999998398	0.9993191919	0.0006806479
32/5	0.9999999978	0.9995868200	0.0004131778
36/5	0.9999999995	0.9997970596	0.0002029398
8	0.9999999995	0.9999136481	0.0000863514

**Table 5:** Comparison between OHAM results given by (53) and numerical solutions for *β* = 1/2.

*η*	*F*_numerical_(*η*)	${\overset{-}{\overset{-}{F}}}_{\text{OHAM}}$(*η*) from (53)	Relative error = $\left|F_{\text{numerical}}(\eta )-{\overset{-}{\overset{-}{F}}}_{\text{OHAM}}(\eta )\right|$
0	−7.585181 · 10^−25^	−2.220446 · 10^−16^	2.220446 · 10^−16^
4/5	0.2543480764	0.2543410815	6.994904 · 10^−6^
8/5	0.8550267840	0.8550382932	0.0000115092
12/5	1.6045273993	1.6045272608	1.384603 · 10^−7^
16/5	2.3963133781	2.3963351657	0.0000217876
4	3.1955002585	3.1955812100	0.0000809514
24/5	3.9954529727	3.9955457399	0.0000927671
28/5	4.7954513949	4.7955738251	0.0001224302
32/5	5.5954513640	5.5956241393	0.0001727753
36/5	6.3954513623	6.3956765240	0.0002251617
8	7.1954513606	7.1957387803	0.0002874196

**Table 6:** Comparison between OHAM results for velocity ${\bar{\bar{F}}}^{\prime }(\eta )$ obtained from (53) and numerical results for *β* = 1/2.

*η*	*F*_numerical_′(*η*)	${{\bar{\bar{F}}}^{}}_{\text{OHAM}}^{\prime }\left(\eta \right)$ from (53)	Relative error = $\left|{F_{\text{numerical}}^{\prime }}_{}\left(\eta \right)-{{\bar{\bar{F}}}^{}}_{\text{OHAM}}^{\prime }\left(\eta \right)\right|$
0	−2.963953 · 10^−20^	0	2.963953 · 10^−20^
4/5	0.5833048175	0.5832372756	0.0000675419
8/5	0.8760975695	0.8761763161	0.0000787466
12/5	0.9760687557	0.9760153463	0.0000534094
16/5	0.9971920744	0.9972855939	0.0000935194
4	0.9998081978	0.9998421267	0.0000339289
24/5	0.9999925759	1.0000087749	0.0000161989
28/5	0.9999998387	1.0000557917	0.0000559529
32/5	0.9999999965	1.0000650747	0.0000650781
36/5	0.9999999980	1.0000682953	0.0000682973
8	0.9999999978	1.0000901947	0.0000901968

**Table 7:** Comparison between OHAM results given by (55) and numerical solutions for *β* = 1/2.

*η*	*F*_numerical_(*η*)	${\overset{-}{\overset{-}{F}}}_{\text{OHAM}}$(*η*) from (55)	Relative error = $\left|F_{\text{numerical}}(\eta )-{\overset{-}{\overset{-}{F}}}_{\text{OHAM}}(\eta )\right|$
0	−5.775724 · 10^−25^	8.881784 · 10^−16^	8.881784 · 10^−16^
4/5	0.2543480764	0.2543611093	0.0000130328
8/5	0.8550267840	0.8550475945	0.0000208105
12/5	1.6045273993	1.6045751046	0.0000477053
16/5	2.3963133781	2.3963685295	0.0000551514
4	3.1955002585	3.1955790615	0.0000788029
24/5	3.9954529727	3.9955715667	0.0001185939
28/5	4.7954513949	4.7956217908	0.0001703959
32/5	5.5954513640	5.5956679550	0.0002165910
36/5	6.3954513623	6.3956963294	0.0002449671
8	7.1954513606	7.1957094147	0.00025805400

**Table 8:** Comparison between OHAM results for velocity ${\bar{\bar{F}}}^{\prime }(\eta )$ obtained from (55) and numerical results for *β* = 1/2.

*η*	*F*_numerical_′(*η*)	${{\bar{\bar{F}}}^{}}_{\text{OHAM}}^{\prime }\left(\eta \right)$ from (55)	Relative error = $\left|{F_{\text{numerical}}^{\prime }}_{}\left(\eta \right)-{{\bar{\bar{F}}}^{}}_{\text{OHAM}}^{\prime }\left(\eta \right)\right|$
0	1.852884 · 10^−21^	−5.329070 · 10^−15^	5.329040 · 10^−15^
4/5	0.5833048175	0.5833475838	0.0000427662
8/5	0.8760975695	0.8761071968	9.627358 · 10^−6^
12/5	0.9760687557	0.9760898679	0.0000211121
16/5	0.99719207446	0.9972074735	0.0000153990
4	0.9998081978	0.9998485848	0.0000403869
24/5	0.99999257599	1.0000520335	0.0000594575
28/5	0.99999983873	1.0000653592	0.0000655205
32/5	0.99999999655	1.0000472907	0.0000472941
36/5	0.99999999803	1.0000244710	0.0000244730
8	0.9999999978	1.0000098270	9.829220 · 10^−6^

**Table 9:** Comparison between the values of ${\bar{F}}^{\prime \prime }(0)$ obtained by means of OHAM and numerical results for *β* = 1/2.

Type of equation	Equation (49)	Equation (51)	Equation (53)	Equation (55)	Numerical
${{\bar{F}}_{\text{OHAM}}^{\prime \prime }}^{}(0)$	0.92767733	0.92763760	0.92760923	0.92779335	0.92768004

**Table 10:** Comparison between OHAM results given by (57) and numerical solutions for *β* = 1.

*η*	*F*_numerical_(*η*)	${\overset{-}{F}}_{\text{OHAM}}$(*η*) from (57)	Relative error = $\left|F_{\text{numerical}}(\eta )-{\overset{-}{F}}_{\text{OHAM}}(\eta )\right|$
0	2.229510 · 10^−25^	−1.110223 · 10^−16^	1.110223 · 10^−16^
4/5	0.3124230332	0.3124218993	1.133942 · 10^−6^
8/5	0.9797795327	0.9797813157	1.783054 · 10^−6^
12/5	1.7552539494	1.7552573134	3.364056 · 10^−6^
16/5	2.5523254690	2.5523466484	0.0000211794
4	3.3521093590	3.3521308223	0.00002146329
24/5	4.1520998358	4.1521444024	0.0000445665
28/5	4.9520995946	4.9522018532	0.0001022586
32/5	5.7520995955	5.7522601900	0.0001605945
36/5	6.5520996012	6.5522991212	0.0001995199
8	7.3520996086	7.3523194836	0.0002198750

**Table 11:** Comparison between OHAM results for velocity ${\bar{F}}^{\prime }\left(\eta \right)$ obtained from (57) and numerical results for *β* = 1.

*η*	*F*_numerical_′(*η*)	${\bar{F}}_{\text{OHAM}}^{\prime }(\eta )$ from (57)	Relative error = $\left|{F_{\text{numerical}}^{\prime }}_{}(\eta )-{\bar{F}}_{\text{OHAM}}^{\prime }(\eta )\right|$
0	−4.599124 · 10^−21^	4.440892 · 10^−16^	4.440938 · 10^−16^
4/5	0.6859374677	0.6859347711	2.696657 · 10^−6^
8/5	0.9323482529	0.9323468816	1.371273 · 10^−6^
12/5	0.9905493983	0.9905683352	0.00001893
16/5	0.9991860373	0.9991967641	0.00001072
4	0.9999584304	0.9999621135	3.683007 · 10^−6^
24/5	0.9999987792	1.0000547905	0.0000560112
28/5	0.9999999836	1.0000795225	0.0000795389
32/5	1.0000000061	1.0000621951	0.0000621890
36/5	1.0000000081	1.0000356813	0.0000356731
8	1.0000000101	1.0000168920	0.0000168818

**Table 12:** Comparison between OHAM results given by (59) and numerical solutions for *β* = 1.

*η*	*F*_numerical_(*η*)	${\overset{-}{F}}_{\text{OHAM}}$(*η*) from (59)	Relative error = $\left|F_{\text{numerical}}(\eta )-{\overset{-}{F}}_{\text{OHAM}}(\eta )\right|$
0	2.229510 · 10^−25^	−2.220446 · 10^−16^	2.220446 · 10^−16^
4/5	0.3124230332	0.3124250905	2.0573489 · 10^−6^
8/5	0.9797795327	0.9797816632	2.1304868 · 10^−6^
12/5	1.7552539494	1.7552458857	8.063655 · 10^−6^
16/5	2.5523254690	2.5523068656	0.0000186033
4	3.3521093590	3.3520844463	0.0000249127
24/5	4.1520998358	4.1520336380	0.0000661977
28/5	4.9520995946	4.9519649131	0.0001346814
32/5	5.7520995955	5.7518965404	0.0002030550
36/5	6.5520996012	6.5518462101	0.0002533911
8	7.3520996086	7.3518163433	0.0002832652

**Table 13:** Comparison between OHAM results for velocity ${\bar{F}}^{\prime }\left(\eta \right)$ obtained from (59) and numerical results for *β* = 1.

*η*	*F*_numerical_′(*η*)	${\bar{F}}_{\text{OHAM}}^{\prime }(\eta )$ from (59)	Relative error = $\left|{F_{\text{numerical}}^{\prime }}_{}(\eta )-{\bar{F}}_{\text{OHAM}}^{\prime }(\eta )\right|$
0	−4.599124 · 10^−21^	1.776356 · 10^−15^	1.776361 · 10^−15^
4/5	0.6859374677	0.6859334525	4.015197 · 10^−6^
8/5	0.9323482529	0.9323592586	0.0000110057
12/5	0.9905493983	0.9905212799	0.0000281183
16/5	0.9991860373	0.9991867807	7.433778 · 10^−7^
4	0.9999584304	0.9999317943	0.0000266361
24/5	0.9999987792	0.9999248393	0.0000739398
28/5	0.9999999836	0.9999090229	0.0000909606
32/5	1.0000000061	0.9999238617	0.0000761443
36/5	1.0000000081	0.9999506141	0.0000493940
8	1.0000000101	0.9999733687	0.0000266414

**Table 14:** Comparison between OHAM results given by (61) and numerical solutions for *β* = 1.

*η*	*F*_numerical_(*η*)	${\overset{-}{\overset{-}{F}}}_{\text{OHAM}}$(*η*) from (61)	Relative error = $\left|F_{\text{numerical}}(\eta )-{\overset{-}{\overset{-}{F}}}_{\text{OHAM}}(\eta )\right|$
0	4.693280 · 10^−25^	6.217248 · 10^−15^	6.217248 · 10^−15^
4/5	0.3124230332	0.3124256050	2.5717950 · 10^−6^
8/5	0.9797795326	0.9797870784	7.545842 · 10^−6^
12/5	1.7552539491	1.7552584440	4.494942 · 10^−6^
16/5	2.5523254682	2.5523369280	0.0000114597
4	3.3521093576	3.3521193533	9.995766 · 10^−6^
24/5	4.1520998332	4.1521125574	0.0000127241
28/5	4.9520995903	4.9521192859	0.0000196955
32/5	5.7520995889	5.7521233093	0.0000237203
36/5	6.5520995917	6.5521296538	0.0000300620
8	7.3520995953	7.3521431339	0.0000435385

**Table 15:** Comparison between OHAM results for velocity ${\bar{\bar{F}}}^{\prime }(\eta )$ obtained from (61) and numerical results for *β* = 1.

*η*	*F*_numerical_′(*η*)	${{\bar{\bar{F}}}^{}}_{\text{OHAM}}^{\prime }\left(\eta \right)$ from (61)	Relative error = $\left|{F_{\text{numerical}}^{\prime }}_{}\left(\eta \right)-{{\bar{\bar{F}}}^{}}_{\text{OHAM}}^{\prime }\left(\eta \right)\right|$
0	−9.390154 · 10^−21^	−4.085620 · 10^−14^	4.085619 · 10^−14^
4/5	0.6859374676	0.6859366849	7.826990 · 10^−7^
8/5	0.9323482527	0.9323490349	7.821535 · 10^−7^
12/5	0.9905493979	0.9905533068	3.908903 · 10^−6^
16/5	0.9991860366	0.9991905903	4.553709 · 10^−6^
4	0.9999584293	0.9999557316	2.697690 · 10^−6^
24/5	0.9999987775	1.0000074611	8.683616 · 10^−6^
28/5	0.9999999812	1.0000067856	6.804426 · 10^−6^
32/5	1.0000000029	1.0000048084	4.805536 · 10^−6^
36/5	1.0000000039	1.0000121725	0.0000121685
8	1.0000000049	1.0000209604	0.0000209554

**Table 16:** Comparison between OHAM results given by (63) and numerical solutions for *β* = 1.

*η*	*F*_numerical_(*η*)	${\overset{-}{\overset{-}{F}}}_{\text{OHAM}}$(*η*) from (63)	Relative error = $\left|F_{\text{numerical}}(\eta )-{\overset{-}{\overset{-}{F}}}_{\text{OHAM}}(\eta )\right|$
0	4.693280 · 10^−25^	0	4.693280 · 10^−25^
4/5	0.3124230332	0.3124174580	5.575244 · 10^−6^
8/5	0.9797795326	0.9797740164	5.516204 · 10^−6^
12/5	1.7552539491	1.7552534223	5.267840 · 10^−7^
16/5	2.5523254682	2.5523611425	0.0000356742
4	3.3521093576	3.3521105770	1.219470 · 10^−6^
24/5	4.1520998332	4.1521005519	7.187418 · 10^−7^
28/5	4.9520995903	4.9521462123	0.0000466219
32/5	5.7520995889	5.7521684524	0.0000688634
36/5	6.5520995917	6.5521510110	0.0000514192
8	7.3520995953	7.3521263893	0.0000267940

**Table 17:** Comparison between OHAM results for velocity ${\bar{\bar{F}}}^{\prime }(\eta )$ obtained from (63) and numerical results for *β* = 1.

*η*	*F*_numerical_′(*η*)	${{\bar{\bar{F}}}^{}}_{\text{OHAM}}^{\prime }\left(\eta \right)$ from (63)	Relative error = $\left|{F_{\text{numerical}}^{\prime }}_{}\left(\eta \right)-{{\bar{\bar{F}}}^{}}_{\text{OHAM}}^{\prime }\left(\eta \right)\right|$
0	−9.390154 · 10^−21^	0	9.390154 · 10^−21^
4/5	0.6859374676	0.6859539897	0.0000165220
8/5	0.9323482527	0.9323092256	0.0000390271
12/5	0.9905493979	0.9906138617	0.0000644637
16/5	0.9991860366	0.9991779292	8.107355 · 10^−6^
4	0.9999584293	0.9999145872	0.0000438420
24/5	0.9999987775	1.0000414108	0.0000426333
28/5	0.9999999812	1.0000539936	0.0000540124
32/5	1.0000000029	0.9999989176	1.085287 · 10^−6^
36/5	1.0000000039	0.9999655183	0.0000344856
8	1.0000000049	0.9999795865	0.0000204184

**Table 18:** Comparison between the values of ${\bar{F}}^{\prime \prime }(0)$ obtained by means of OHAM and numerical results for *β* = 1.

Type of equation	Equation (57)	Equation (59)	Equation (61)	Equation (63)	Numerical
${{\bar{F}}_{\text{OHAM}}^{\prime \prime }}^{}(0)$	1.23258247	1.23257895	1.23262084	1.23257391	1.232558769

**Table 19:** Comparison between OHAM results given by (65) and numerical solutions for *β* = 1.6.

*η*	*F*_numerical_(*η*)	${\overset{-}{F}}_{\text{OHAM}}$(*η*) from (65)	Relative error = $\left|F_{\text{numerical}}(\eta )-{\overset{-}{F}}_{\text{OHAM}}(\eta )\right|$
0	−1.692327 · 10^−25^	−1.110223 · 10^−16^	1.110223 · 10^−16^
4/5	0.3599784956	0.3599782796	2.160525 · 10^−7^
8/5	1.0696147641	1.0696156734	9.093019 · 10^−7^
12/5	1.8571623264	1.8571633242	9.977941 · 10^−7^
16/5	2.6560434964	2.6560463745	2.878145 · 10^−6^
4	3.4559804211	3.4559769848	3.436309 · 10^−6^
24/5	4.2559782714	4.2559782610	1.038556 · 10^−8^
28/5	5.0559782427	5.0559829283	4.685640 · 10^−6^
32/5	5.8559782660	5.8559798484	1.582358 · 10^−6^
36/5	6.6559783023	6.6559717863	6.516027 · 10^−6^
8	7.4559783545	7.4559638500	0.0000145044

**Table 20:** Comparison between OHAM results for velocity ${\bar{F}}^{\prime }(\eta )$ obtained from (65) and numerical results for *β* = 1.6.

*η*	*F*_numerical_′(*η*)	${\bar{F}}_{\text{OHAM}}^{\prime }(\eta )$ from (65)	Relative error = $\left|{F_{\text{numerical}}^{\prime }}_{}(\eta )-{\bar{F}}_{\text{OHAM}}^{\prime }(\eta )\right|$
0	4.071713 · 10^−20^	9.992007 · 10^−16^	9.991600 · 10^−16^
4/5	0.7609225381	0.7609240247	1.486624 · 10^−6^
8/5	0.9619780339	0.9619706813	7.352687 · 10^−6^
12/5	0.9960567187	0.9960657372	9.018419 · 10^−6^
16/5	0.9997439284	0.9997365151	7.413383 · 10^−6^
4	0.9999899725	0.9999872482	2.724280 · 10^−6^
24/5	0.9999997837	1.0000081444	8.360731 · 10^−6^
28/5	1.0000000206	1.0000013591	1.338446 · 10^−6^
32/5	1.0000000368	0.9999918129	8.223935 · 10^−6^
36/5	1.0000000541	0.9999891905	0.0000108635
8	1.0000000764	0.9999914106	8.665844 · 10^−6^

**Table 21:** Comparison between OHAM results given by (67) and numerical solutions for *β* = 1.6.

*η*	*F*_numerical_(*η*)	${\overset{-}{F}}_{\text{OHAM}}$(*η*) from (67)	Relative error = $\left|F_{\text{numerical}}(\eta )-{\overset{-}{F}}_{\text{OHAM}}(\eta )\right|$
0	−1.692327 · 10^−25^	1.332267 · 10^−15^	1.332267 · 10^−15^
4/5	0.3599784956	0.3599798414	1.345740 · 10^−6^
8/5	1.0696147641	1.0696168199	2.055868 · 10^−6^
12/5	1.8571623264	1.8571595718	2.754588 · 10^−6^
16/5	2.6560434964	2.6560360629	7.433444 · 10^−6^
4	3.4559804211	3.4559769853	3.435805 · 10^−6^
24/5	4.2559782714	4.2559486488	0.0000296226
28/5	5.0559782427	5.0558929543	0.0000852884
32/5	5.8559782660	5.8558366505	0.0001416154
36/5	6.6559783023	6.6557973327	0.0001809696
8	7.4559783545	7.4557756153	0.0002027391

**Table 22:** Comparison between OHAM results for velocity ${\bar{F}}^{\prime }(\eta )$ obtained from (67) and numerical results for *β* = 1.6.

*η*	*F*_numerical_′(*η*)	${\bar{F}}_{\text{OHAM}}^{\prime }(\eta )$ from (67)	Relative error = $\left|{F_{\text{numerical}}^{\prime }}_{}(\eta )-{\bar{F}}_{\text{OHAM}}^{\prime }(\eta )\right|$
0	4.071713 · 10^−20^	−3.552713 · 10^−15^	3.552754 · 10^−15^
4/5	0.7609225381	0.7609190201	3.517984 · 10^−6^
8/5	0.9619780339	0.9619872725	9.238582 · 10^−6^
12/5	0.9960567187	0.9960397833	0.0000169354
16/5	0.9997439284	0.9997513373	7.408827 · 10^−6^
4	0.9999899725	0.9999817723	8.200210 · 10^−6^
24/5	0.9999997837	0.9999433979	0.0000563857
28/5	1.0000000206	0.9999242317	0.0000757889
32/5	1.0000000368	0.9999386977	0.0000613391
36/5	1.0000000541	0.9999628614	0.0000371926
8	1.0000000764	0.9999814328	0.0000186436

**Table 23:** Comparison between OHAM results given by (69) and numerical solutions for *β* = 1.6.

*η*	*F*_numerical_(*η*)	${\overset{-}{\overset{-}{F}}}_{\text{OHAM}}$(*η*) from (69)	Relative error = $\left|F_{\text{numerical}}(\eta )-{\overset{-}{\overset{-}{F}}}_{\text{OHAM}}(\eta )\right|$
0	−1.692327 · 10^−25^	−1.693090 · 10^−15^	1.693090 · 10^−15^
4/5	0.3599784956	0.3599790235	5.278046 · 10^−7^
8/5	1.0696147640	1.0696154385	6.744980 · 10^−7^
12/5	1.8571623261	1.8571642418	1.915749 · 10^−6^
16/5	2.6560434955	2.6560445435	1.048024 · 10^−6^
4	3.4559804190	3.4559816246	1.205614 · 10^−6^
24/5	4.2559782671	4.2559820737	3.806680 · 10^−6^
28/5	5.0559782344	5.0559813610	3.126620 · 10^−6^
32/5	5.8559782515	5.8559788900	6.385224 · 10^−7^
36/5	6.6559782783	6.6559788961	6.177966 · 10^−7^
8	7.4559783175	7.4559820586	3.74109314 · 10^−6^

**Table 24:** Comparison between OHAM results for velocity ${\bar{\bar{F}}}^{\prime }(\eta )$ obtained from (69) and numerical results for *β* = 1.6.

*η*	*F*_numerical_′(*η*)	${{\bar{\bar{F}}}^{}}_{\text{OHAM}}^{\prime }\left(\eta \right)$ from (69)	Relative error = $\left|{F_{\text{numerical}}^{\prime }}_{}\left(\eta \right)-{{\bar{\bar{F}}}^{}}_{\text{OHAM}}^{\prime }\left(\eta \right)\right|$
0	−2.832266 · 10^−21^	2.664535 · 10^−15^	2.664538 · 10^−15^
4/5	0.7609225380	0.7609232141	6.760967 · 10^−7^
8/5	0.9619780338	0.9619796540	1.620229 · 10^−6^
12/5	0.9960567183	0.9960575197	8.014067 · 10^−7^
16/5	0.9997439274	0.9997418375	2.089891 · 10^−6^
4	0.9999899704	0.9999929170	2.946522 · 10^−6^
24/5	0.9999997799	1.0000016372	1.857256 · 10^−6^
28/5	1.0000000145	0.9999969829	3.031551 · 10^−6^
32/5	1.0000000272	0.9999978766	2.150573 · 10^−6^
36/5	1.0000000399	1.0000022282	2.188252 · 10^−6^
8	1.0000000667	1.0000052427	5.176061 · 10^−6^

**Table 25:** Comparison between OHAM results given by (71) and numerical solutions for *β* = 1.6.

*η*	*F*_numerical_(*η*)	${\overset{-}{\overset{-}{F}}}_{\text{OHAM}}$(*η*) from (71)	Relative error = $\left|F_{\text{numerical}}(\eta )-{\overset{-}{\overset{-}{F}}}_{\text{OHAM}}(\eta )\right|$
0	−1.692327 · 10^−25^	−2.331468 · 10^−15^	2.331468 · 10^−15^
4/5	0.35997849	0.3599784037	9.195208 · 10^−8^
8/5	1.06961476	1.0696144385	3.254580 · 10^−7^
12/5	1.85716232	1.8571634346	1.108560 · 10^−6^
16/5	2.65604349	2.6560430260	4.694645 · 10^−7^
4	3.455980419	3.4559810382	6.192629 · 10^−7^
24/5	4.25597826	4.2559845299	6.262826 · 10^−6^
28/5	5.05597823	5.0559832266	4.992205 · 10^−6^
32/5	5.85597825	5.8559812915	3.039980 · 10^−6^
36/5	6.65597827	6.6559890626	0.0000107842
8	7.4559783175	7.4560065554	0.0000282378

**Table 26:** Comparison between OHAM results for velocity ${\bar{\bar{F}}}^{\prime }(\eta )$ obtained from (71) and numerical results for *β* = 1.6.

*η*	*F*_numerical_′(*η*)	${{\bar{\bar{F}}}^{}}_{\text{OHAM}}^{\prime }\left(\eta \right)$ from (71)	Relative error = $\left|{F_{\text{numerical}}^{\prime }}_{}\left(\eta \right)-{{\bar{\bar{F}}}^{}}_{\text{OHAM}}^{\prime }\left(\eta \right)\right|$
0	−2.832266 · 10^−21^	3.552713 · 10^−15^	3.552716 · 10^−15^
4/5	0.7609225380	0.7609232919	7.538631 · 10^−7^
8/5	0.9619780338	0.9619761488	1.885029 · 10^−6^
12/5	0.9960567183	0.9960594353	2.717000 · 10^−6^
16/5	0.9997439274	0.9997398342	4.093197 · 10^−6^
4	0.9999899704	0.9999971751	7.204662 · 10^−6^
24/5	0.9999997799	1.0000032843	3.504355 · 10^−6^
28/5	1.0000000145	0.9999951986	4.815848 · 10^−6^
32/5	1.0000000272	1.0000024525	2.425327 · 10^−6^
36/5	1.0000000399	1.0000168296	0.0000167896
8	1.0000000667	1.0000253832	0.0000253165

**Table 27:** Comparison between the values of ${\overset{-}{\overset{-}{F}}}^{\prime \prime }(0)$ obtained by means of OHAM and numerical results for *β* = 1.6.

Type of equation	Equation (65)	Equation (67)	Equation (69)	Equation (71)	Numerical
${\overset{-}{\overset{-}{F}}}_{\text{OHAM}}^{\prime \prime }(0)$	1.52151589	1.52151820	1.52151245	1.52151403	1.52151402
